# A Numerical Simulation Study of the Impact of Kesterites Hole Transport Materials in Quantum Dot-Sensitized Solar Cells Using SCAPS-1D

**DOI:** 10.3390/nano14242016

**Published:** 2024-12-15

**Authors:** Sindisiwe Jakalase, Azile Nqombolo, Edson L. Meyer, Mojeed A. Agoro, Nicholas Rono

**Affiliations:** 1 Fort Hare Institute of Technology, University of Fort Hare, Private Bag X1314, Alice 5700, Eastern Cape, South Africa; 202016188@ufh.ac.za (S.J.); emeyer@ufh.ac.za (E.L.M.); magoro@ufh.ac.za (M.A.A.); 2 Department of Chemistry, University of Fort Hare, Private Bag X1314, Alice 5700, Eastern Cape, South Africa; anqombolo@ufh.ac.za

**Keywords:** renewable energy, QDSSCs, kesterites, numerical simulation, SCAPS-1D

## Abstract

Energy generation and storage are critical challenges for developing economies due to rising populations and limited access to clean energy resources. Fossil fuels, commonly used for energy production, are costly and contribute to environmental pollution through greenhouse gas emissions. Quantum dot-sensitized solar cells (QDSSCs) offer a promising alternative due to their stability, low cost, and high-power conversion efficiency (PCE) compared to other third-generation solar cells. Kesterite materials, known for their excellent optoelectronic properties and chemical stability, have gained attention for their potential as hole transport layer (HTL) materials in solar cells. In this study, the SCAPS-1D numerical simulator was used to analyze a solar cell with the configuration FTO/TiO_2_/MoS_2_/HTL/Ag. The electron transport layer (ETL) used was titanium dioxide (TiO_2_), while Cu_2_FeSnS_4_ (CFTS), Cu_2_ZnSnS_4_ (CZTSe), Cu_2_NiSnS_4_ (CNTS), and Cu_2_ZnSnSe_4_ (CZTSSe) kesterite materials were evaluated as HTLs. MoS_2_ quantum dot served as the absorber, with FTO as the anode and silver as the back metal contact. The CFTS material outperformed the others, yielding a PCE of 25.86%, a fill factor (FF) of 38.79%, a short-circuit current density (J_SC_) of 34.52 mA cm^−2^, and an open-circuit voltage (V_OC_) of 1.93 V. This study contributes to the advancement of high-performance QDSSCs.

## 1. Introduction

In recent decades, the global demand for energy has been experiencing a significant rise due to rapid industrialization, trade openness, and urbanization [1]. Currently, most countries are depending on fossil fuels such as coal, gas, and oil to generate power, but this has posed severe pressure on their availability [2]. The reliance on fossil fuels by many nations for the production of energy generates greenhouse gases that can disturb the atmosphere and global climate. As such, research is focused on the identification of alternative clean sources of energy that can maintain environmental integrity [3]. Renewable energy is an acceptable alternative to fossil fuels due to its cleanliness and wide applicability in many various fields [4]. Presently, scientists have devoted much effort to exploring renewable energy systems and making them affordable, sustainable, and more efficient [5]. Renewable energy sources include hydrogen, solar, biomass, geothermal, and wind energies [6]. Among these energy sources, solar energy is more environmentally [6] and economically viable than other renewable energy sources due to its cleanliness [7], accessibility [8,9], and inexhaustibility [10].

Solar energy is one of the most inexpensive and efficient energy sources that is being explored and used today [11,12]. Interest in solar cells has increased significantly as a newly developed device to meet environmental sustainability and the energy shortage challenge [13]. Solar cells are categorized into three generations, namely, first-, second-, and third-generation, based on power conversion efficiency (PCE), time, and the nature of the materials utilized to make them [14,15]. Some of the examples of the aforementioned three-generation solar cells include silicon-based [16], thin film-based [17], organic solar cells (OSCs) [18], perovskite solar cells (PSCs) [19], dye-sensitized solar cells (DSSCs) [20], and quantum dot-sensitized solar cells (QDSSCs) [21]. Third-generation solar cells, particularly QDSSCs, have emerged as attractive candidates because of the ease of fabrication, low cost, and the use of environmentally friendly materials [22].

Thus, QDSSCs are considered a promising low-cost alternative to existing photovoltaic technologies that use quantum dots (QDs) as the absorber materials [23]. Quantum dot materials exhibit tunable bandgaps, which are suitable in quantum dot-sensitized cells, therefore increasing the efficiency of solar cells [24]. Research on QDSSCs is currently in progress, aiming to address challenges such as stability and cost-effectiveness. Basically, QDSSCs have three major components, that is, a photoanode, a counter electrode, and an electrolyte (active layer) [24,25]. In comparison, ternary organic solar cells (ternary OSCs) also offer a simplified fabrication process with a single-layer structure, which reduces manufacturing complexity [26]. While ternary OSCs can achieve improved efficiency by incorporating a third component into the active layer, they may face limitations in long-term stability and efficiency under various environmental conditions. Perovskite solar cells, another emerging technology, offer high efficiency and relatively low manufacturing costs [27], with the potential for scalability [28]. However, they suffer from challenges related to material stability and the toxicity of lead-based components. Dye-sensitized solar cells (DSSCs), while offering flexibility and transparency, have lower efficiency compared to QDSSCs [29]. Additionally, DSSCs face challenges related to long-term stability, particularly in outdoor conditions, due to degradation of the dye and electrolyte. Hence, QDSSCs have been chosen for this study.

Quantum dots are getting attention in display technology owing to their remarkable optical properties such as high efficiency, high absorption coefficients, lower fabrication cost, tunable band gaps, and precise emission wavelength [30,31,32]. Quantum dots are essential materials in solar cell research since they enable researchers to explore possibilities beyond the restrictions of traditional photovoltaics (PVs) [33,34]. Their electronic and optical properties are governed by quantum mechanics due to their small sizes (typically the particle sizes range between 1 and about 10 nm) [35,36]. In general, QDs have utilized diverse modern technologies, including light-emitting diodes (LEDs), photodetectors, photovoltaics, biomedicine, and the environment [37]. Quantum dots (QDs) have found significant applications in display technologies, particularly in quantum-dot light-emitting diodes (QLEDs) [38]. These displays are widely used in products such as Samsung’s QLED TVs, which utilize the unique optical properties of QDs to enhance color accuracy and brightness. The application of QDs in liquid crystal displays (LCDs) has been particularly transformative, enabling more vibrant colors and better energy efficiency. A comprehensive review by Chen et al. [39] provides detailed insights into these advancements, demonstrating the importance of QDs in modern display technology. This study highlights the growing role of quantum dots in commercial products, further emphasizing their versatility beyond photovoltaics and their integration into consumer electronics.

From the literature, QDSSCs have several shortcomings, including insufficient optimization of interface properties, toxicity concerns associated with certain materials, the need for more efficient and stable hole transport materials, inadequate modeling and simulation of tools, etc. [40,41]. There is a need to develop high-efficiency, stable, and scalable QDSSC architecture by addressing the aforementioned challenges through innovative materials, interfaces, and device engineering that will eventually result in a commercially viable technology.

Numerical simulation has become a popular method for a comprehensive understanding of solar cell technologies, including organic solar cells (OSCs) [42], perovskites (PSCs) [43], dye-sensitized solar cells (DSSCs) [20], and QDSSCs [44]. Numerical simulation offers several key advantages when it comes to solar cell design and analysis. It allows for the testing of a wide range of materials, providing flexibility in exploring various options without the need for costly and time-consuming physical experimentation [45]. Moreover, numerical simulations provide deeper insights into the underlying device physics, revealing details that might be challenging or impossible to access through experimental methods alone. This combination of cost-effectiveness, time efficiency, and enhanced understanding makes numerical simulation an invaluable tool in the development of advanced solar cell technologies.

Different types of software have been used in simulation, such as Silvaco ATLAS [46], general-purpose photovoltaic device model (GPVDM) [47], wx-Analysis of Microelectronic and Photonic Structures (wxAMPSs) [48], and Simulator Cell Capacitance Simulator (SCAPS-1D) [49,50]. Wahid et al. [51] reported a thin-film solar cell (TFSC) based on molybdenum disulfide (MoS_2_) with an integrated copper(I) oxide (Cu_2_O) hole transport layer (HTL) utilizing the SCAPS-1D software. Their cell configuration was as follows: Al/ZrS_2_/ZnO/MoS_2_/Cu_2_O/Ni, and they exhibited a PCE of 26.70%, with an FF of 80.85%, J_SC_ of 30.33 mA cm^−2^, and a V_OC_ of 1.089 V. Moustafa et al. [52] conducted a study on numerical analysis of the CZTS-based solar cell SCAPS-1D using the following cell configuration: ZnO (AZO)/ZrS_2_/CZTS. The device achieved a PCE of 17.61%, an FF of 84.75%, a J_SC_ of 27.75 mA cm^−2^, and a V_OC_ of 0.776 V.

Herein, we report a numerical simulation of a cell with the following configuration: FTO/TiO_2_/MoS_2_/kesterite/Ag using SCAPS-1D software (version 3.3.10). The proposed and tested HTL-based kesterite materials were CFTS, CZTSe, CNTS, and CZTSSe, while TiO_2_ was used as a common ETL for all the devices and MoS_2_ as an absorber. The devices with different HTL materials were then optimized. Kesterite materials were proposed because they are non-toxic, abundant, and cost-effective materials with a tunable band gap, high hole mobilities, and significant absorption coefficients, making them suitable alternatives as HTL in solar cells. In essence, the influence of varying the density defect (N_t_) of the absorber and the doping density of ETL (N_D_), using different HTL materials (kesterites) and TiO_2_ as ETL, on the performance was investigated. The influence of the variation of ETL, HTL, and absorber layer thicknesses was also investigated. Furthermore, the effect of altering the band gap of the absorber layer, metallic back contact, and operation temperature on overall device performance has been investigated. It is anticipated that the findings of this study will be beneficial for the future fabrication of highly efficient QDSSCs. QDSSCs have demonstrated significant progress in recent years, but further improvement is necessary to achieve high stability and scalability. Further research and development are crucial to overcoming existing limitations, paving the way for commercialization and widespread adoption of QDSSCs as a viable solution for renewable energy generation.

## 2. Materials and Methods

**Figure 1:**
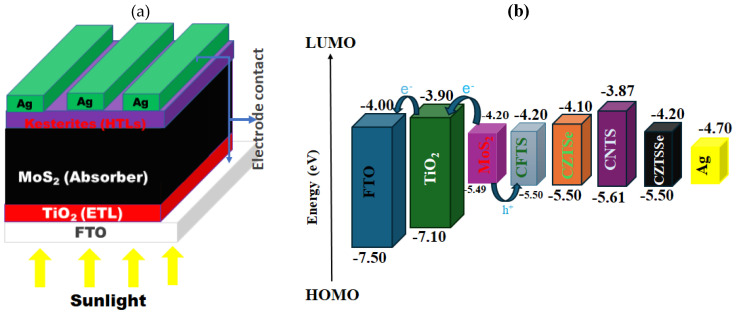
(**a**) Solar cell device architecture and (**b**) the band alignment between the absorber, the proposed HTLs, and the metallic back contact (Ag).

The proposed solar cell (FTO/TiO_2_/MoS_2_/kesterite/Ag) was modeled and simulated using the Simulator Cell Capacitance Simulator (SCAPS-1D) software and is represented in Figure 1a. The device was made up of several layers stacked together, that is, FTO as a counter electrode, MoS_2_ used as an absorber, TiO_2_ as an ETL, four kinds of kesterite materials (CFTS, CZTS, CNTS, and CZTSe) as HTLs, and Ag as a counter electrode. Kesterites were proposed over other materials because they offer significant advantages in terms of sustainability [53]. Unlike other materials used in solar cells, such as lead-based perovskites, kesterites are composed of non-toxic [54,55], abundant elements like copper, zinc, and tin, which make them an environmentally friendly alternative [55]. The use of these elements reduces concerns related to material scarcity and toxicity, which are critical factors in the widespread adoption of solar technology. In contrast, materials like cadmium telluride (CdTe), although efficient, present environmental challenges due to the toxicity of cadmium [56,57]. Furthermore, kesterites are potentially more cost-effective [58], as the raw materials are widely available and relatively inexpensive compared to other semiconductors. While the production processes for kesterite-based solar cells may still involve energy-intensive steps, the overall environmental impact is expected to be lower than that of more complex or toxic materials. Therefore, kesterite materials may not only offer a competitive edge in terms of efficiency but also contribute to the development of more sustainable and environmentally responsible solar technologies [55]. For the simulation, the solar spectrum was set at an air mass of 1.5 global (AM 1.5G), working temperature of 300 K, and the power density of 1000 W m^−2^. The corresponding band alignment of various components of the device is shown in Figure 1b.

The SCAPS-1D numerical simulator was used in the computational analysis of modeled solar cells with different kesterite HTL materials. SCAPS-1D is a one-dimensional simulation software used to generate various solar cell architectures developed and implemented by Prof. Marc Burgelman with his research students at the University of Gent [59]. The software can be used to model a device with up to seven different layers and thus can be used to also model tandem solar cells. The input parameters of the defect interfaces of the modeled device were carefully extracted from published studies. They are presented in Table 1 and Table 2, respectively. Silver metal was considered a back metal contact, and its work function was 4.7 eV [60].

The control panel of the software allows users to set up the simulation conditions, such as light intensity, light spectrum, temperature, frequency, shunt resistance, series resistance, and mesh points, for detailed analysis. After the cell has been modeled and conditions defined, its output characterization is performed using SCAPS by solving Poisson’s equation. The software is based on the semiconductor equations at steady states, that is, the Poisson, continuity, and the diffusion and drift of electrons and holes equations [67]. The relationship between electric field (E) and space charge density can be expressed in Equation (1) below:

(1) $$\frac{\partial^{2}\psi }{\partial^{2}x}=-\frac{\partial E}{\partial X}=-\frac{\rho }{\varepsilon_{s}}=-\frac{q}{\varepsilon_{s}}[p-n+N_{D}^{+}\left(x\right)-N_{A}^{-}\left(x\right)\pm N_{def}(x)]$$

where ψ denotes the electrostatic potential, $\varepsilon_{s}$ is the relative static permittivity of free space, q is the elementary charge, n is the electron density, p is the hole density, $N_{D}^{+}$ is the density of the ionized donors, $N_{A}^{-}$ is the density of ionized acceptors, and N_def_ is the defect density of the acceptor or donor [68].

Equations (2) and (3) represent the equations of continuity for electrons and holes, respectively.

(2) $$\frac{{\partial j}_{n}}{\partial x}+G-Un(n,p)=0$$

(3) $$-\frac{{\partial j}_{p}}{\partial x}+G-Up(n,p)=0$$

where j_n_ stands for the electron current density, j_p_ is the hole current density, U_n,p_ is the net recombination rate, and G is the generation rate. Equations (4) and (5) express how the charge carriers in the device move by diffusion and drift for the electrons and holes, respectively, as follows:

(4) $$j_{n}=D_{n}\;\frac{dn}{dx}+\mu_{n}\;n\;\frac{d\emptyset }{dx}$$

(5) $$j_{p}=D_{n}\frac{dn}{dx}+\mu_{p}\;p\;\frac{d\emptyset }{dx}$$

where D_n_ is the electron diffusion coefficient, µ_n_ is the electron mobility, D_p_ is the hole diffusion coefficient, µ_p_ is the hole mobility, and ∅ is the electrostatic potential [69].

While SCAPS-1D was the primary software used for this analysis, other simulation software could also be employed to validate the results or offer additional insights, for instance, the ATLAS simulation software [70]. Similarly, wxAMPS [71] (developed by Washington State University) is another valuable tool for simulating the electrical properties of semiconductor devices, particularly in the context of multi-junction or tandem solar cells. wxAMPS provides detailed simulations of current–voltage characteristics, ideal for investigating the effects of material composition and layer thicknesses on performance. Other notable potential simulation software includes PC1D [72], which is designed for simulating the performance of solar cells under varying light conditions, particularly useful for understanding the impact of different illumination intensities on the device efficiency. Additionally, the Sentaurus TCAD software [73] is a powerful tool for simulating semiconductor devices, including solar cells. It can model carrier transport, recombination, and electrical characteristics across various materials and device configurations, providing a comprehensive view of solar cell performance. By incorporating these simulation tools into future studies, researchers can cross-validate findings from SCAPS-1D and gain deeper insights into the performance and behavior of solar cells, providing a more comprehensive and reliable understanding of device characteristics.

## 3. Results and Discussion

### 3.1. Impact of the Utilization of Different Hole Transport Layers

In order to validate the result of our simulation, calibration of SCAPS-1D was first carried out by extrapolation from previous studies. In essence, calibration of the SCAPS-1D software involves adjusting its parameters to match experimental or real-world data, ensuring that the simulation accurately reflects the performance of the solar cell under various conditions. Bencherif et al. [74] successfully performed experimental validation of SCAPS-1D by comparing the experimental and the obtained theoretical values, and it was established that it was very close, thus demonstrating that the software gave accurate and reliable results. Additionally, Mattaparthi et al. [75] demonstrated that their experimental PCE was comparable with the theoretically achieved results by using SCAPS-1D. It is also worth mentioning that there exists a slight deviation from the experimental result to the theoretical one, attributed to the fact that the SCAPS-1D model may not be affected by the normal environmental conditions.

In this section, a numerical simulation was used to examine the impact of the utilization of different HTLs in the device with the general architecture of FTO/TiO_2_/MoS_2_/HTL/Ag on the performance and theoretical stability. Thickness adjustment is a crucial step in improving the performance of solar cells; hence the FTO, HTL, absorber, and ETL thicknesses for each of the devices with varied HTLs were fine-tuned [76]. It involved systematically adjusting the thickness of the layers in the solar cell to achieve optimal performance metrics such as PCE, FF, V_OC_, and J_SC_. The HTL materials tested included CFTS, CZTSe, CNTS, and CZTSSe. In essence, adjustment was performed repeatedly by altering the thickness of an individual layer while keeping the thickness of the other layers constant. Each layer was studied sequentially until a maximum PCE was attained. For the device with CFTS HTL material, the thickness alteration was executed as follows: the thickness of HTL was varied from 1.600 to 2.200 µm until a maximum PCE was attained at 1.900 µm, while the thicknesses of FTO, ETL, and an absorber remained constant at 0.100, 0.500, and 0.200 µm, respectively. The thickness of CFTS was then fixed, and in the meantime, FTO and MoS_2_ were kept constant also, while the thickness of ETL was varied from 0.001 to 0.500 µm until the maximum value of PCE was obtained at 0.001 µm. The thickness variation of the absorber layer was from 0.010 to 0.200 µm; nevertheless, the absorber, HTL, and FTO thicknesses remained fixed. The optimal absorber thickness was determined to be 0.020 µm. Lastly, the FTO thickness was optimized by altering it between 0.010 and 0.060, with 0.030 µm being the best thickness. A similar optimization approach was applied to all of the devices simulated in this study using different initial thicknesses for each layer. The result of the adjusted thicknesses of each device is presented in Table 3.

In essence, the current–voltage curve (J-V characteristics) demonstrates how a solar cell’s output of current is related to the applied voltage [58]. The J-V properties of a solar cell can be affected by several factors, including temperature, incident light intensity, and the kind of solar cell. The V_OC_ of each device was achieved through the choice of HTL material that influenced the energy band alignment with the absorber layer, facilitating efficient hole extraction and minimizing energy losses. By optimizing the HTL properties, such as mobility and conductivity, charge recombination at the interface was reduced, therefore allowing for higher V_OC_.

**Figure 2:**
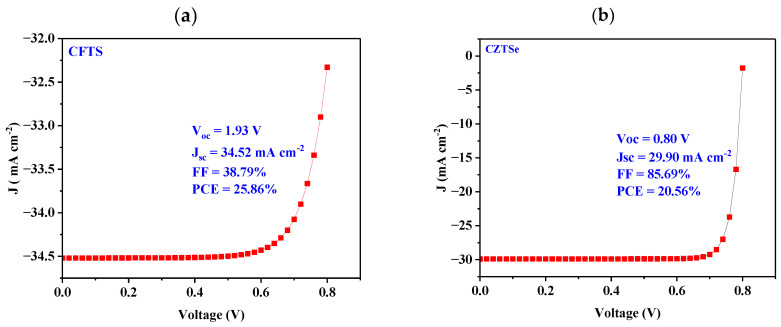

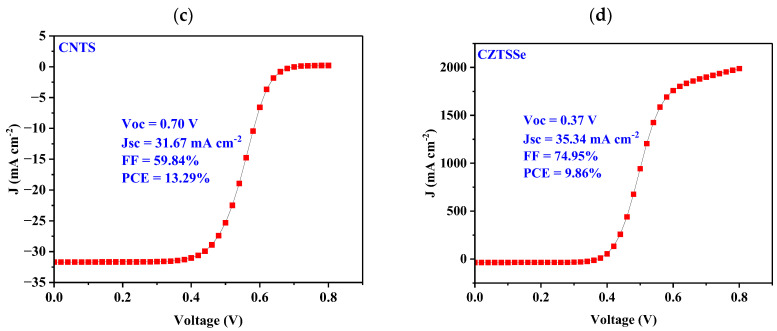
J-V curves of (**a**) CFTS-, (**b**) CZTSe-, (**c**) CNTS-, and (**d**) CZTSSe-based devices.

Figure 2 displays the J-V characteristic for solar cells with varied HTL materials and their corresponding performances. CFTS, a ternary chalcogenide compound, has emerged as a promising material for solar cells due to its advantageous properties, making it a strong candidate for photovoltaic applications [77]. Remarkably, the CFTS-based device (Figure 2a) exhibits the highest PCE of 25.86% amongst all the kesterite candidates. The other photovoltaic metrics, namely, FF, J_SC_, and V_OC_ for the best device were found to be 38.79%, 34.52 mA cm^−2^, and 1.93 V, respectively. The best achieved performance of the CFTS-based device was ascribed to proper band alignment of an absorber and a high absorption coefficient in the visible to near-infrared range to absorb light efficiently. It was also attributed to good hole mobility and low recombination losses, which help in efficient charge transport and collection within the solar cell. In comparison to polymer solar cells, Cu_2_FeSnS_4_ (CFTS) shows promising advantages in terms of stability and material sustainability. Recent advancements in organic solar cells, as highlighted in the study [78], have led to efficiencies exceeding 18%. However, while organic solar cells offer flexibility and ease of fabrication, they often face challenges related to long-term stability, particularly under environmental stress such as exposure to moisture and UV light. However, CFTS, with its earth-abundant and non-toxic composition, provides a more stable [77] and sustainable alternative, with the potential for lower-cost production. CFTS also avoids the use of toxic materials, such as lead or cadmium, commonly found in other high-efficiency solar cells, further enhancing its environmental appeal. While the efficiency of CFTS solar cells is still improving, their inherent stability, sustainability, and the availability of non-toxic raw materials position them as strong candidates for next-generation solar technologies.

It was also observed that CZTSe HTL material showed a relatively higher PCE of 20.56% (Figure 2b), while CNTS HTL material (Figure 2c) achieved a relatively low PCE of about 13.29%. From Figure 2d, it is evident that the CZTSSe-based device exhibited the lowest PCE of 9.86% when compared to all other tested materials. This could be attributed to increased recombination of the photogenerated charges due to ineffective band alignment, thus lowering the performance.

**Figure 3:**
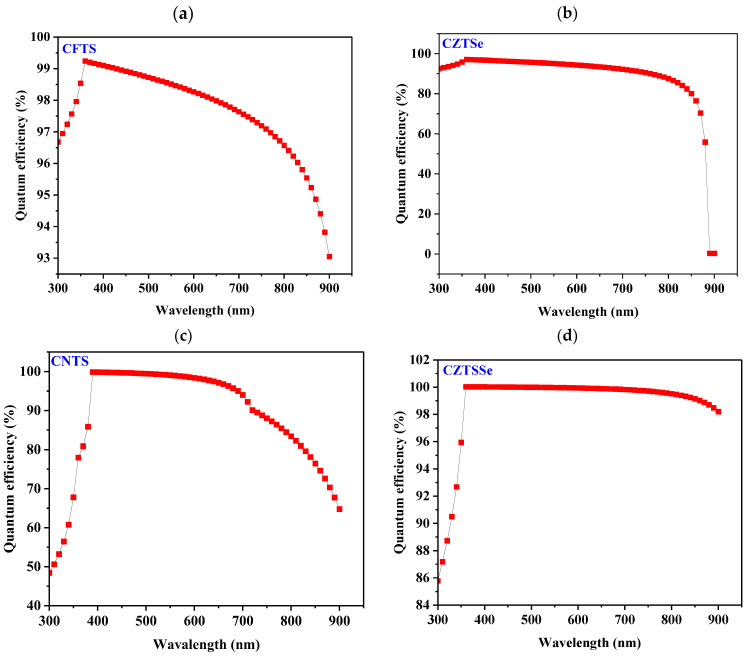
Quantum efficiencies of (**a**) CFTS-, (**b**) CZTSe-, (**c**) CNTS-, and (**d**) CZTSSe-based devices.

Quantum efficiency (QE) is a significant component in solar cell applications that measures how effective a device is in converting incident photons into electrons. Here, the QE values for optimized devices with various kesterite HTL materials have been simulated, and the results are graphically presented in Figure 3. The illumination wavelength range was between 300 and 900 nm for this study. Under ideal conditions, the QE for a specific wavelength is always one. CFTS- and CZTSe-based devices, as shown in Figure 3a,b, respectively, had a remarkably high QE of above 90% from 300 to 700 nm, meaning that the devices could absorb both infrared and visible light radiation. QE decreased significantly for devices with CFTS, CZTSe, and, to some extent, CNTS as HTL materials (Figure 3c). These devices could not absorb light towards infrared regions, and thus, QE decreased. Notably, the CZTSSe-based device (Figure 3d) showed no significant change throughout the measured visible region. It was also noted that all kesterites showed different curves, as shown in Figure 3, which implies that, although the HTL is located at the back of the device, much light passes through them, hence the different QE curves. This could be attributed to the relatively thinner FTO, ETL, and absorber layers used and some transparency of the components (Table 2). In essence, the obtained QE results highlight strong performance across a broad spectrum, including the visible and near-infrared regions. This wide spectral response could have significant implications for practical applications, particularly in indoor photovoltaics. Indoor environments often rely on artificial lighting, such as LEDs and fluorescent lamps, which emit light primarily in the blue and red regions of the spectrum [79]. Given the strong QE response of kesterite-based solar cells in these regions, they show promise for efficiently capturing light from such sources, making them potential candidates for low-light energy harvesting applications. Although this study focuses on the characterization of the quantum efficiency from simulated solar spectrum (AM 1.5G), these findings suggest that kesterite-based devices could be effective in powering small electronic devices, sensors, or energy-efficient systems in indoor settings, where the available light intensity is much lower than outdoors.

Moreover, since the obtained QE curves provide valuable insights into the wavelength regions where the device absorbs light most effectively, understanding the QE profile allows for the optimization of absorber materials and device architectures to enhance light harvesting, particularly in the most critical regions of the spectrum. By examining the QE results, experimentalists can fine-tune material selection and layer configurations to achieve maximum absorption efficiency, guiding decisions on the choice of semiconductor materials or additional coatings that can boost performance.

### 3.2. Optimization of Absorber Layer (MoS_2_) Thickness

**Figure 4:**
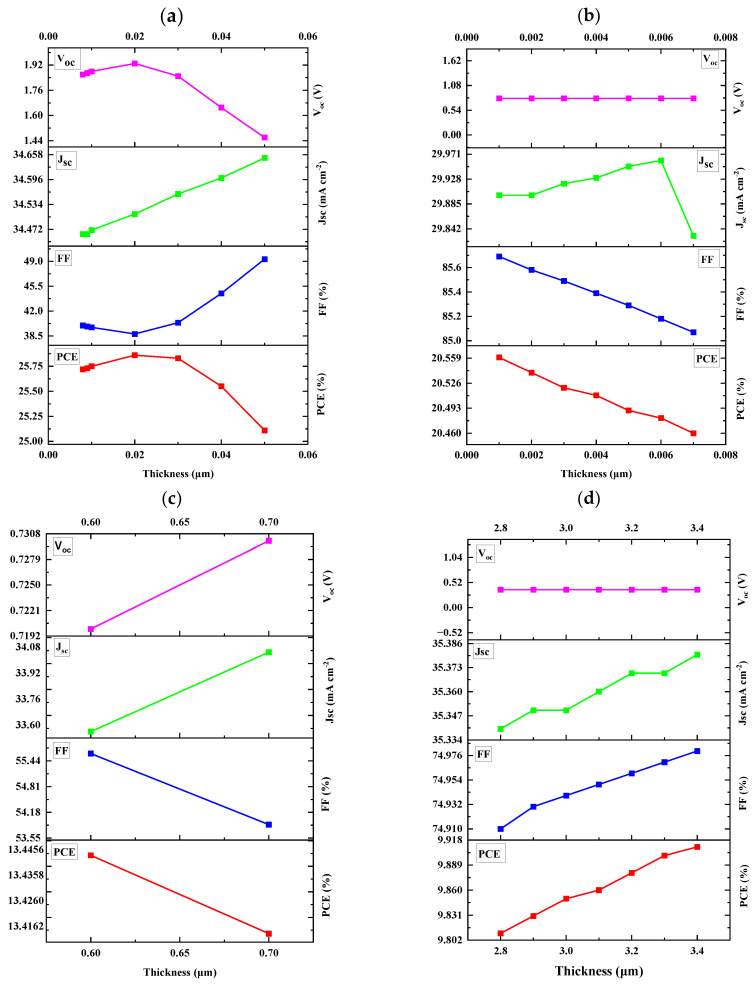
MoS_2_ thickness variation with respect to PCE, FF, J_SC_, and V_OC_ in different HTL materials (**a**) CFTS-, (**b**) CZTSe-, (**c**) CNTS-, and (**d**) CZTSSe-based devices.

The thickness of the absorber layer was modified in different ranges depending on the HTL material that was used, while the ETL and HTL layers remained unmodified. The thicknesses of the absorbers in the CFTS-, CZTSe-, CNTS-, and CZTSSe-based devices were modified within the ranges of 0.008 to 0.050 µm, 0.001 to 0.007 µm, 0.100 to 0.700 µm, and 2.800 to 3.400 µm, respectively. It is important to note that for the CFTS-based device (Figure 4a), both the PCE and V_OC_ decreased as the thickness of the absorber increased, while FF and J_SC_ were increasing. For instance, the PCE decreased from 25.72 to 25.11% and V_OC_ decreased from 1.86 to 1.46 V. The reason behind the decrease in PCE and V_OC_ may be due to the fact that thicker absorbers can lead to higher rates of charge recombination, ultimately leading to a reduction in both V_OC_ and PCE. Although a thicker layer may seem beneficial for light absorption, it may also hinder the effective transport of charge carriers to the surface, therefore reducing their collection efficiency. An increase in FF and J_SC_ may be attributed to a thicker layer that might reduce defects, contributing to better charge transport and higher FF. Figure 4b also revealed a decrease in efficiency as the thickness of the absorber was increasing, which might be assigned for the same reason mentioned above. The V_OC_ remained constant at 0.80 V throughout; the stability trend observed during the thickness variation can be attributed to the consistent generation of electron–hole pairs. The same findings of the constant V_OC_ were obtained by Yagoub and Adnane [80]. As the thickness of the MoS_2_ layer increased, it maintained its ability to facilitate the same number of electron–hole pairs reaching the active layer. This phenomenon highlights the reliable nature of electron–hole pair generation across the entire thickness range.

For the CNTS-based device (Figure 4c), as the thickness of the absorber layer decreased, both the PCE and FF showed a decreasing trend, while J_SC_ and V_OC_ increased. The decrease in PCE and FF may be due to compromised charge transport in very thin layers, potentially resulting in increased resistive losses. Thinner absorbers may enhance light penetration and absorption efficiency, contributing to a higher J_SC._ The observed increase in V_OC_ might be due to the shorter distance charge carriers that must travel to reach the collection interface, which can lead to reduced recombination losses and therefore result in an increase in V_OC_. It can also be noted that the CNTS-based device exhibited photovoltaic parameters only at the thickness of 0.60 to 0.70 µm; outside of this range, no photovoltaic parameters were observed. The CZTSSe-based solar cell (Figure 4d) demonstrated an increase in PCE, FF, and J_SC_ as the thickness of the absorber layer increased, while V_OC_ remained constant as the thickness of the absorber layer increased. This trend suggests that the thicker absorber layer effectively enhanced the overall performance of the cell without negatively impacting the voltage output [80]. Experimentally, thickness variation is crucial for optimizing the absorption of light and charge carrier collection [81,82]. Thinner absorber layers may reduce material usage and manufacturing costs but could also lead to less efficient light absorption, while thicker layers might improve absorption but introduce issues such as increased recombination or reduced charge transport efficiency. Thus, the thickness optimization process provides a guideline for selecting an optimal absorber thickness that balances these competing factors, hence, guiding experimental fabrication by identifying thickness values that maximize device efficiency while maintaining manageable material costs and ensuring stable performance [83]. Besides absorber thickness optimization, multilayer or tandem solar cell architectures offer another promising approach for enhancing performance [84]. Tandem solar cells, by stacking multiple absorber layers with complementary bandgaps, can increase light absorption across a wider spectral range, resulting in higher overall efficiency. Recent research, such as the study [85], has highlighted the potential of using advanced materials like corroles in tandem configurations, which can help capture a broader portion of the solar spectrum and improve energy conversion efficiency. Incorporating similar strategies into kesterite-based devices could further enhance performance, positioning them as a competitive option for next-generation solar technologies.

### 3.3. HTL Material Thickness Optimization

The thicknesses of different HTL materials were optimized, as illustrated in Figure S1 in the supplementary document. The thicknesses for CFTS-, CZTSe-, CNTS-, and CZTSSe-based devices were modified within the ranges of 1.60 to 2.2 µm, 2.10 to 2.70 µm, 0.30 to 0.90 µm, and 1.60 to 2.20 µm, respectively. Figure S1a results revealed that PCE, J_SC_, and V_OC_ exhibited an increase as the thickness of the CFTS layer increased, reaching their maximum at 25.94%, 34.58 mA cm^−2^, and 1.95 V, respectively, while FF showed a decreasing trend. The V_OC_ increase can be attributed to thicker layers that can make it easier to generate more electron–hole pairs. Thicker layers might also introduce challenges in charge transport, resulting in a lower FF despite improvements in J_SC_ and V_OC_. However, the decrease in FF may be attributed to increased charge recombination rates as the thickness increases, which can hinder efficient charge collection. Figure S1b also demonstrated an increase in PCE and J_SC_ alongside a decrease in FF values as the thickness of the CZTSe-based device was increasing. The PCE increased from 20.16 to 20.88%, whereas J_SC_ increased from 29.47 to 30.23 mA cm^−2^ for the thicknesses ranging from 2.1 to 2.7 µm. The V_OC_ was constant at 0.79 V for thicknesses between 2.1 and 2.2 µm, then increased at 2.3 µm to 1.80 V, after which it stabilized.

Figure S1c showed an increase in PCE and J_SC_ values as the thickness of the CNTS layer increased. The PCE increased from 13.22 to 13.29%, while J_SC_ increased from 31.51 to 31.66 mA cm^−2^ in the thickness range of 0.3 to 0.6 µm. The V_OC_ remained constant at 0.70 V. This stability trend exhibited during the thickness variation may be attributed to the consistent generation of electron–hole pairs. The FF values increased from 59.75 to 59.87% as the thickness increased from 0.30 to 0.50 µm and then decreased to 59.84% at 0.60 µm. It can also be observed that other thicknesses did not show any photovoltaic parameters. Figure S1d demonstrated an increase in PCE and J_SC_ and a decrease in FF values as the thickness of the CZTSSe-based device increased. The V_OC_ was increased from 0.36 to 0.37 V for thicknesses between 1.60 and 2.20 µm and then remained constant throughout. The decrease in FF values may be related to increased resistive losses at greater thickness.

### 3.4. Influence of ETL (TiO_2_) Thickness in Different HTL Materials

This study entailed modifications to the thickness of ETL (TiO_2_), which is crucial to solar cell performance. In the CFTS-, CZTSe-, CNTS-, and CZTSSe-based devices, the absorber thicknesses were adjusted between 0.001 and 0.007 µm, 0.001 and 0.007 µm, 0.300 and 0.900 µm, and 0.001 and 0.007 µm, respectively, while other layers were kept unchanged, as presented in Table 1. Remarkably, significant changes were observed in various solar cell devices, including V_OC_, FF, J_SC_, and PCE. Notably, PCE, J_SC_, and V_OC_ exhibited constant values at 25.86%, 34.51 mA cm^−2^, and 1.93 V, respectively, across the range of TiO_2_ thickness variations in Figure S2a. The observed results indicate that changes in the thickness of TiO_2_ have a limited impact on these three photovoltaic parameters. It suggests that the thickness of the ETL has a minimal effect on charge carrier recombination. The FF increased from 38.79 to 38.80%, from 0.001 to 0.003 µm, and remained constant throughout. Figure S2b also revealed a constant value of 0.80 V for V_OC_, but other parameters were changing. The J_SC_ increased from 29.90 to a constant value of 29.89 mA cm^−2^. The FF revealed a decreasing trend, while PCE decreased slightly from 20.56 to 20.54%. These findings may be attributed to the increased absorption of light within the TiO_2_, which may lead to a reduced availability of photons for the absorber layer. As a result, the generation of electron–hole pairs decreased, consequently contributing to a reduction in overall efficiency and charge transport.

Figure S2c showed that the FF and V_OC_ remained constant at 59.84% and 0.70 V, respectively, at thicknesses of 0.60 and 0.70 µm. The constant FF and V_OC_ may indicate that the potential barrier is optimized at these thicknesses. However, the PCE was decreasing while the J_SC_ was increasing. The increase in J_SC_ could be attributed to enhanced light absorption at greater thickness, allowing more charge carriers to be generated. The decrease in PCE may indicate that the device was experiencing higher recombination losses, which could hinder overall efficiency. It was also noted that other thicknesses did not show any photovoltaic parameters. Figure S2d displayed constant values of 0.37 V, 35.36 mA cm^−2^, 74.95%, and 9.86% for V_OC_, J_SC_, FF, and PCE, respectively. The device design might be optimized for these specific metrics, resulting in consistent performance across all the measured parameters.

### 3.5. Defect Density of an Absorber

**Figure 5:**
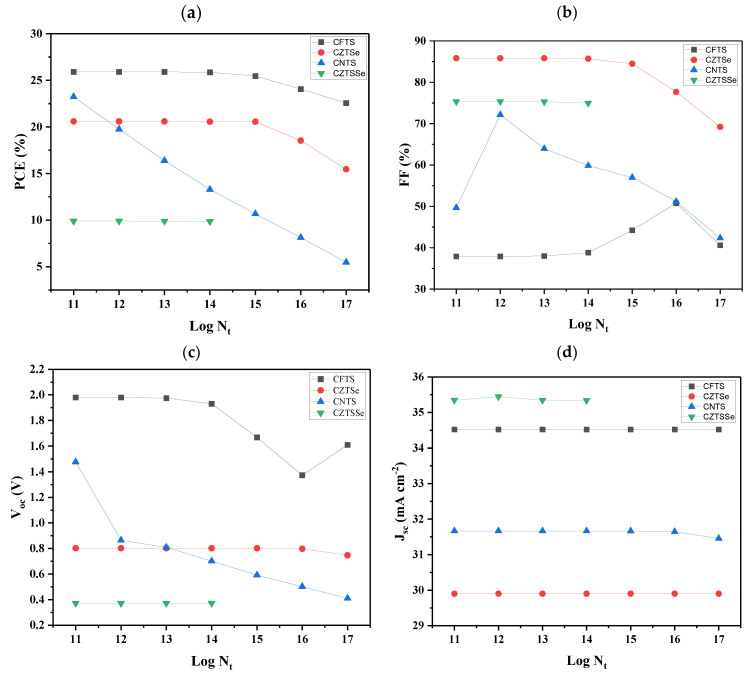
Variation of photovoltaic parameters for devices by changing defect density of an absorber in a range of 1 × 10^11^ to 1 × 10^17^ for devices with different HTLs: (**a**) PCE, (**b**) FF, (**c**) V_oc_, and (**d**) J_sc._

The density defect of an absorber has a significant impact on the PV properties of the device [86]. Defects in a poor-quality absorber lead Shockley-Read-Hall non-radiative recombination centers to form, delaying the arrival of excitons at the terminals. This affects the performance of the device significantly [87]. To investigate the effect of defect density on absorber performance, the density of defects (N_t_) was varied from 1 × 10^11^ to 1 × 10^17^ cm^−3^ for devices with different HTL materials. The resulting characteristics are shown in Figure 5. In Figure 5a, it can be observed that the PCE of the CFTS-based device was found to be generally higher when compared to other devices. The PCEs of CFTS- and CZTSe-based devices remained constant from the 11th to the 15th order, and they decreased in the 16th and 17th order. This indicates that the CFTS- and CZTSe-based devices were more tolerant of higher levels of defects. For the CNTS-based device, the PCE sharply decreased as the density of defects increased, while for the CZTSSe-based device, the PCE was constant from the 11th to 14th order, and it did not show any photovoltaic behavior for the remaining orders, attributed to increased charge recombination.

Figure 5b revealed that the CZTSe-based device had the highest FF values for the 11th to the 15th order levels of defect density, and then it decreased from the 16th to the 17th order. Meanwhile, the CZTSSe-based device had constant FF values from the 11th to the 14th order, and it also did not show for the remaining orders. The FF for the CNTS-based device increased from around 48 to above 70% in the 11th to the 12th order and then decreased throughout the remaining orders ascribed to increased recombination of photogenerated charges. CFTS-based devices had the lowest FF values, which were constant in the 11th to the 15th order compared to other solar cell devices in this study. It was also observed that the FF increased from the 14th to the 16th order and then decreased in the 17th order. However, the V_OC_ graph (Figure 5c) showed the highest values of V_OC_ for the CFTS-based device in all orders. The V_OC_ for the CZTSe-based device was constant from the 11th to the 16th order, and it decreased in the 17th order. The V_OC_ values were decreasing throughout all the orders. Lastly, for the CZTSSe-based device, it was constant from the 11th to 14th orders, and it did not show results for other orders. From Figure 5d, it can be observed that the CZTSSe-based device had the highest J_SC_ values for all the orders, followed by CFTS-, CNTS-, and lastly CZTSe-based devices. It was also observed that they were constant throughout, except for the 17th order of the CNTS-based device, which slightly decreased. It was also noted that the V_OC_ increased slightly in the 12th order of the CZTSSe-based device. It can be concluded that high defect density levels can lead to a high rate of recombination of photogenerated charges and hence lead to decreased solar cell performance. It is, therefore, crucial to maintain the lowest possible level of defects without compromising the layer’s quality. Recent studies have demonstrated the importance of interface engineering in enhancing the defect tolerance and performance of solar cells. For example, structural modulation techniques, such as those described in the study [88], have been used to improve the morphology and crystallinity of solar cell layers, reducing defects at the interfaces. Applying similar strategies to kesterite-based materials, particularly at the interface between the kesterite absorber and hole transport layers, could help minimize defects, enhance charge transport, and improve the overall stability and efficiency of the devices. This approach highlights the potential of interface engineering as a key factor in optimizing the performance of kesterite solar cells.

### 3.6. Effect of Doping the ETL Donor Density

**Figure 6:**
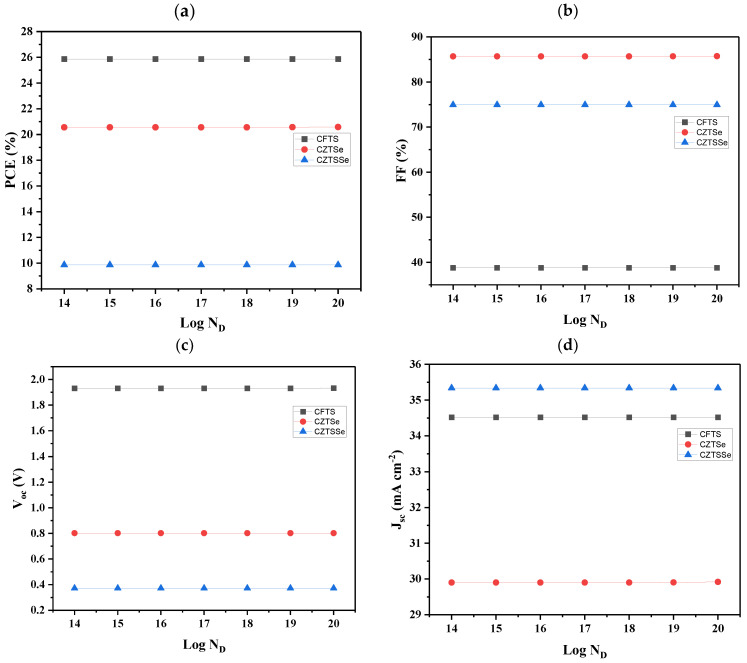
Effect of variation of ETL donor density from 1 × 10^14^ to 1 × 10^20^ cm^−3^ of devices with TiO_2_ as the ETL and different HTLs: (**a**) PCE, (**b**) FF, (**c**) V_oc_, and (**d**) J_sc_.

The effect of doping the ETL was also examined by varying the donor density doping levels from 14th to 20th order. The results are presented in Figure 6. Figure 6 shows that there was no change in photovoltaic parameters for all devices as the donor density of ETLs was varied. The PCE (Figure 6a) was found to be 25.86, 20.56, and 9.86% for CFTS-, CZTSe-, and CZTSSe-based devices, respectively. Figure 6b revealed the constant values of FF, which were 85.69, 74.97, and 38.79% for CZTSe-, CZTSSe-, and CFTS-based devices, respectively, for all orders. A higher V_OC_ generally means that the cell can generate a higher voltage potential, which is crucial for achieving higher power output. Figure 6c proved the aforementioned statement; here, the V_OC_ values were also constant at all doping levels, with the CFTS-based device having the highest values, followed by the CZTSe-based device and then the CZTSSe-based device. The J_SC_ values (Figure 6d) were found to be constant for all the doping levels of N_D_, and they were 35.33, 34.51, and 29.90 mA cm^−2^. The observed constant values might be due to the device parameters, which may be more influenced by other dominated factors such as the quality of the absorber layer, interface properties, or other materials. As a result, variations in ETL donor density may have a minimal impact on overall performance. Moreover, the electric field within the device plays a critical role in charge separation and transport. If the electric field strength remains unchanged across varying donor densities, the device performance may not vary significantly. When varying the ETL donor density of the cell with CNTS as the HTL material, the cell did not run at all doping levels, probably due to the detrimental effects of excessive doping levels.

### 3.7. The Effect of Varying Temperature

**Figure 7:**
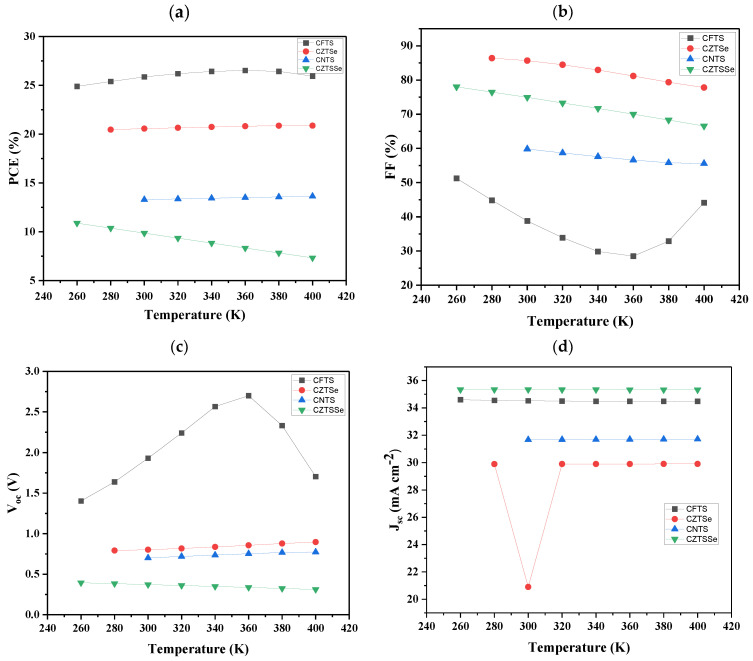
The influence of temperature of the devices containing TiO_2_ as ETL, MoS_2_ as an absorber, and different HTL materials: (**a**) PCE, (**b**) FF, (**c**) V_oc_, and (**d**) J_sc_.

The external temperature affects the performance of the solar cells and their structural stability. The operating temperature gradient influences the cell layer properties, including carrier concentrations, the effective density of states, electron and hole mobility, absorption coefficients, electron and hole thermal velocity, and material bandgap [89]. The numerical simulation of the solar cell devices investigated in this study was performed at a temperature of 300 K; nonetheless, the simulation was performed at different temperatures to determine the effect of utilizing different temperatures in each device. At the starting temperature of 240 K, devices did not show any photovoltaic behavior; therefore, to investigate the effect of the temperature on the absorber performance using different HTL materials for all the devices, the temperature was then varied from 260 to 400 K. The main reason for simulating these cells over this temperature range is because the solar panels are normally exposed outside of the buildings, for instance, on top of the roof or windows. The resulting photovoltaic characteristics for the devices with different HTL materials are illustrated in Figure 7.

Meanwhile, higher temperatures have been established to cause the greatest impact on the V_OC_ parameter and thus, the efficiency of a solar cell [90]. From Figure 7a, the device with CFTS as the HTL material showed higher PCEs, which were between the range of 24.89 and 26.52% for all the optimized temperatures, followed by CZTSe with the PCEs of around 20%, then CNTS with the PCEs, which were around 13%. Lastly, CZTSSe with the PCEs between the range of 7.33 and 10.38%. The results obtained for each cell indicated that the operation of the cell is affected by the temperature changes. It can be observed (Figure 7a) that as the temperature was increasing, the PCE values of devices were increasing except for the CZTSe-based device. Similar results were obtained by [61,91]. This might be due to the fact that higher temperatures can increase the thermal energy of charge carriers, improving their mobility. This can lead to more efficient transport charges within the device, enhancing the overall performance of the devices. For the device with CZTSSe as the HTL material, the PCE decreased with increasing temperature. The decrease in PCE for the CZTSSe-based device as a function of increasing temperature was ascribed to the fact that an increase in temperature leads to increased kinetic energy of charges, thus leading to more collisions and reducing their transport. Additionally, at a higher temperature, the band gap is reduced, leading to more recombination of excitons; thus a decrease in PCE is observed as the operating temperature increases. Several reports have shown the similar effect of temperature on the solar cell performance [60,92,93].

The FF parameter decreased as the temperature increased for CFTS, CZTSe, and CNTS-based devices (Figure 7b), except for the CZTSSe device, which decreased gradually from 260 to 360 K and then increased from 380 to 400 K. Additionally, it was observed that the device with CZTSe as the HTL material had the highest FFs, followed by the device with CZTSSe, then CNTS, and lastly CFTS. The V_OC_ of the devices (Figure 7c) with CZTSe and CNTS as the HTL materials increased as the temperature was increasing and vice versa with the one containing CZTSSe. The V_OC_ of the devices with CFTS increased from 260 to 360 K, and then it decreased for the rest of the temperatures. Moreover, it was pointed out that the device with CFTS had the highest V_OC_ compared to others.

According to Chander et al. [94], J_SC_ is proportional to the quantity of photogenerated charge carriers, which also increases with the operating temperature in solar cells. However, this is not the case in this study, where J_SC_ remains constant for other temperatures. The J_SC_ (Figure 7d), however, remained constant for the devices with CFTS as the HTL material. For the device having CNTS as the HTL material, the photovoltaic parameters at 260 and 280 K did not show, but showed at 300 to 400 K, and then remained constant for temperatures from 300 to 400 K. CZTSe solar cell did not show any change in J_SC_ for all the tested operational temperatures except for 300 K, which experienced an unusual dip. However, the J_SC_ generally decreases slightly from 29.90 to 20.90 mA cm^−2^. For the CZTSSe-based device, the Jsc decreased between a temperature of 260 to 360 K, and then it became constant. Notably, input parameters such as band gap, charge carrier concentration, and mobility of holes and electrons are susceptible to changes in the operational temperature of any electronic device, including solar cells.

Additionally, the reason J_SC_ remains constant can be explained by understanding the underlying physics of the photogeneration and collection processes within the solar cell. First, J_SC_ is directly proportional to the number of photogenerated electrons and holes in the solar cell. When the cell is exposed to light, photons with energy equal to or greater than the absorber material’s bandgap form electron–hole pairs. The built-in electric field of the p-n junction or heterojunction separates these pairs, which are subsequently collected as current. If the solar cell is irradiated at a constant light intensity, the rate of electron–hole pair creation remains constant. As a result, J_SC_ remains constant because it is directly proportional to the quantity of photons absorbed and the efficiency with which charge carriers separate. At short-circuit conditions, when the voltage across the solar cell is zero, the current is exclusively driven by photogenerated carriers and is not impacted by the external circuit [83].

While temperature fluctuations can significantly influence the efficiency and charge transport of kesterite-based solar cells, it is also essential to evaluate their long-term stability under real-world conditions. Long-term tests, including temperature cycling, humidity exposure, and UV degradation, are crucial for understanding how these devices will perform over time in outdoor environments and ensuring their viability for commercial applications. For example, previous studies have shown that materials like kesterite may experience performance degradation over time due to thermal stress and moisture absorption, which can lead to the breakdown of the absorber layer or interfaces. Therefore, including stability tests under simulated real-world conditions, such as temperature cycling, humidity exposure, and UV degradation, will provide valuable insights into the durability of kesterite-based solar cells and their potential for long-term deployment in outdoor environments.

### 3.8. Effect of Bandgap Variation

**Figure 8:**
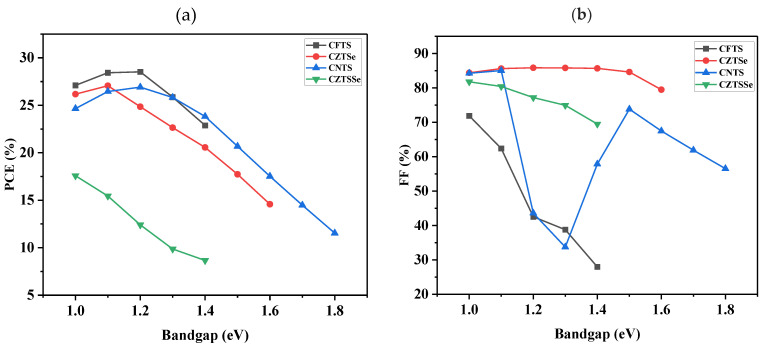

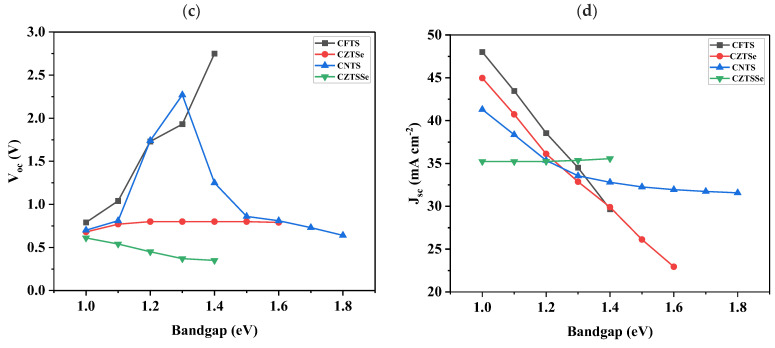
The influence of bandgap energy of the devices containing TiO_2_ as ETL, MoS_2_ as an absorber, and different HTL materials: (**a**) PCE, (**b**) FF, (**c**) V_oc_, and (**d**) J_sc._

Figure 8 illustrates the photovoltaic parameters versus bandgap energy graphs for various HTL materials in a simulated FTO/TiO_2_/MoS_2_/Kesterites/Ag solar cell. The bandgap was systematically varied from 1.00 to 1.90 eV across all devices. As expected, the behavior of CFTS-, CZTSe-, and CNTS-based devices follows the typical pattern in solar cells; a low energy bandgap provides a high short-circuit current density, which slowly decreases as the bandgap increases. Figure 1a showed a decrease in PCE of the devices as the bandgap energy decreases. Reyes et al. [93] also obtained similar results. The decrease in PCE may be attributed to the fact that higher bandgap materials tend to absorb less of the solar spectrum. This reduced absorption results in lower photon capture and, consequently, diminishes the current generation. Figure 8b illustrates a decrease in FF values as the bandgap energy increases for CFTS-, CZTSe-, and CZTSSe-based devices, while the CNTS-based device exhibited fluctuation. The reasons for the decrease in FF values may be assigned to changes that might happen in carrier mobility at higher bandgaps, which can influence the transport of charges, affecting the fill factor in the devices.

Figure 8c showed an increase in V_OC_ on CFTS- and CZTSe-based devices as the bandgap energy was increasing, whereas for the CZTSSe-based device, it was decreasing. The behavior of the CNTS-based device, which shows an increase in voltage from 0.70 to 0.86 V as the bandgap energy rises from 1.0 to 1.5 eV, followed by a decrease to 0.64 V, might be attributed to an increase in bandgap, which might improve carrier generation and transport, contributing to higher voltage. However, at higher bandgap values, carrier mobility might decrease, leading to a reduction in voltage. In Figure 8d, it is observed that the J_SC_ generally decreases with increasing bandgap energy across most devices. However, the CZTSSe-based device exhibited an increase in J_SC_ as the bandgap energy increased. Higher bandgap materials tend to absorb a narrower range of the solar spectrum. As the bandgap increases, the ability to capture lower-energy photons decreases, resulting in lower overall current generation. The CZTSSe material may have a favorable absorption spectrum that allows it to capture more light, even at higher bandgap energies, leading to an increase in J_SC_. It can also be noted that within the bandgap range of 1.5 to 1.9 eV, the photovoltaic parameters did not show CFTS- and CZTSSe-based devices. Similarly, for the CZTSe-based device between 1.7 and 1.9 eV and the CNTS-based device at 1.9 eV, no notable photovoltaic parameters were observed.

### 3.9. Effect of Varying Metal Back Contact

The photovoltaic performance of solar cells has been established to be significantly influenced by the type of back contact and metal work function [95]. Silver is a preferable anode material for investigating ohmic contact behavior and rectifying properties and is mostly studied by researchers. The appropriate built-in voltage is demonstrated by the back-contact material to set an appropriate work function. In this study, five different back-contact materials, including aluminum (Al), gold (Au), copper (Cu), molybdenum (Mo), and selenide (Se) with various work functions, have been explored. The obtained PCE are shown in Table 4. The work functions for Al, Au, Cu, Mo, and Se were 4.26, 5.47, 5.10, 4.95, and 5.90 eV [96], respectively. It was observed that as the metal work function of back contacts was increasing, the PCE increased for all of the HTL materials. The reason behind the increment in PCE was ascribed to a high work function, which supports the attraction of holes. For CNTS- and CZTSSe-based devices, the efficiencies increased rapidly compared to CFTS- and CZTSe-based devices. The PCE for the CFTS-based device was found to be 27.87% for all back metal contacts except Al, which was found to be 22.23%. For other kesterite materials, the PCE did not show when Al was used as the metal back contact. In addition, the PCE of the CZTSSe-based device was found to be the lowest when Ag back metal was used, but it was noted that when Au was used, the PCE became higher than that of the CZTSe- and CNTS-based devices. It was also observed that when Se was used, the PCE was higher compared to the CNTS-based device. In general, other relatively cheaper metals (Cu, Mo, and Se) than gold can be used as alternative back contacts. In general, our reported power conversion efficiency (PCE) of 25.86% for CFTS-based devices represents a significant achievement in comparison to the current state-of-the-art performance of quantum dot-sensitized solar cells (QDSSCs), which have achieved PCEs in the range of 15–17% in recent studies. While QDSSCs have made notable advancements in terms of efficiency, their performance is often limited by issues such as poor charge transport and the instability of quantum dot materials. In contrast, CFTS-based devices demonstrate superior stability and efficiency due to the robust, earth-abundant, and non-toxic nature of kesterite materials. The novelty of our approach lies in the integration of kesterite-based materials like CFTS into solar cells, which are not only cost-effective but also exhibit high stability, outperforming many current materials used in QDSSCs and being a cost-effective solution compared to other high-efficiency solar cell technologies. This study highlights the potential of kesterite-based devices as a promising alternative to QDSSCs, offering both high efficiency and long-term stability for next-generation solar energy applications. For further comparison purposes, other results obtained from both numerical simulation and experiment compared to our simulated models are presented in Table 5.

## 4. Conclusions

In this study, the potential application of four kesterite candidates (CFTS, CZTSe, CNTS, and CZTSSe) as potential HTLs was explored using the SCAPS-1D numerical simulator. TiO_2_ was used as the ETL, MoS_2_ acted as the absorber, FTO was used as the photoelectrode, and Ag as the counter electrode. The results demonstrated the PCE of 25.86, 20.56, 13.29, and 9.86% for CFTS-, CZTSe-, CNTS, and CZTSSe-based devices, respectively. The highest PCE of the CFTS-based device was attributed to its superior material properties, such as narrow band gap and better charge carrier mobility, which contribute to higher efficiency in energy conversion. The density of defects of the absorber on performance was also investigated; the density defect varied from 1 × 10^11^ to 1 × 10^17^ cm^−3^, and the optimal density of defect was kept at 1 × 10^15^ cm^−3^. Nonetheless, it was also noted that the PCE of the devices decreased as the density defect of an absorber material increased. The effect of doping the ETL donor density was also examined by varying the doping levels from 1 × 10^11^ to 1 × 10^17^ cm^−3^. It was observed that the PCE, FF, J_SC_, and V_OC_ remained constant for each cell throughout, but for the cell with CNTS as the HTL material, it did not show any photovoltaic parameters, probably due to detrimental effects of over-doping, leading to high recombination of charges.

The effect of variation in temperature was also examined between the range of 240 and 400 K. The PCE of the devices increased as the temperature was increasing except for the CZTSSe-based device. The results obtained were attributed to the higher temperatures that may have increased the thermal energy of charge carriers, improved their mobility, and enhanced charge transport, which boosted the overall device performance. In contrast, for the CZTSSe-based device, the decrease in performance could be attributed to the rise in temperature, which increases the kinetic energy of charge carriers. This leads to more frequent collisions, ultimately reducing the efficiency of charge transport within the device. The band gap variation was also examined, and the results showed a decrease in PCE of the devices as the bandgap energy decreases. The decrease in PCE may be attributed to the fact that materials with a higher bandgap absorb less of the solar spectrum, limiting the amount of light that can be converted into electrical energy. It was observed that as the metal work function of the back contact increased, the PCE increased for all tested devices. Other devices, except with CFTS, did not show any photovoltaic performance when Al metal was used as a back contact, attributed to its relatively low work function. The results of this study are envisaged to propel the commercialization of MoS_2_-based solar cells.

## Figures and Tables

**Table 1:** The base input parameters used in the simulation for this study.

Parameters	FTO [61]	TiO_2_ [62]	MoS_2_ [63]	CFTS [64]	CZTSe [64]	CNTS [64]	CZTSSe [65]
**Band gap, Eg (eV)**	3.50	3.20	1.29	1.30	1.40	1.74	1.30
**Electron affinity, χ (eV)**	4.00	3.90	4.20	4.20	4.10	3.87	4.20
**Dielectric Permittivity, e_r_**	9.00	32.00	20.00	3.00	9.00	9.00	13.60
**Density of states at CB, N_c_ (cm^–3^)**	2.2 × 10^18^	1.0 × 10^19^	2.2 × 10^18^	2.2 × 10^18^	2.2 × 10^18^	2.2 × 10^18^	2.2 × 10^18^
**Density of states at VB, N_v_ (cm^–3^)**	1.8 × 10^19^	1.0 × 10^19^	1.8 × 10^19^	1.8 × 10^19^	1.8 × 10^19^	1.8 × 10^19^	1.8 × 10^19^
**Electron mobility, μ_e_ (cm^2^ V^−1^s^−1^)**	20.00	20.00	1.0 × 10^2^	21.98	100.00	11.00	100.00
**Hole mobility, μ_h_ (cm^2^ V^−1^s^−1^)**	10.00	10.00	1.5 × 10^2^	21.98	12.50	11.00	25.00
**Density n-type doping, *N*_D_ (cm^–3^)**	1.0 × 10^19^	1.0 × 10^17^	1.0 × 10^14^	0.00	0.00	0.00	1.0 × 10^1^
**Density p-type doping, *N*_A_ (cm^–3^)**	0.00	0.00	1.0 × 10^15^	1.0 × 10^19^	1.0 × 10^19^	1.0 × 10^19^	1.0 × 10^15^
**Defect density, N_t_ (cm^−3^)**	0.00	1.0 × 10^16^	1.0 × 10^14^	1.0 × 10^14^	1.0 × 10^14^	1.0 × 10^14^	1.0 × 10^13^ [66]

**Table 2:** Input parameters for the defect interfaces.

Interface Parameter	HTL/Active Layer	ETL/Active Layer
**Defect type**	Neutral	Neutral
**Capture cross-section electrons (cm^2^)**	1.0 × 10^−19^	1.0 × 10^−19^
**Capture cross-section holes (cm^2^)**	1.0 × 10^−19^	1.0 × 10^−19^
**Energetic distribution**	Single	Single
**Reference for defect energy level E_t_**	Above the highest E_V_	Above the highest E_V_
**Energy with respect to a reference (eV)**	0.600	0.600
**Interface defect (cm^−2^)**	Variable	Variable

**Table 3:** Optimized thicknesses for different layers of a device using TiO_2_ as an ETL, MoS_2_ as an absorber, and different HTLs.

Cell Configuration	FTO (μm)	ETL (μm)	Absorber (μm)	HTL (μm)
**FTO/TiO_2_/MoS_2_/CFTS/Ag**	0.030	0.001	0.020	1.900
**FTO/TiO_2_/MoS_2_/CZTSe/Ag**	0.050	0.001	0.001	2.400
**FTO/TiO_2_/MoS_2_/CNTS/Ag**	0.200	0.600	0.400	0.600
**FTO/TiO_2_/MoS_2_/CZTSSe/Ag**	0.100	0.001	3.100	1.900

**Table 4:** The effect of using different metal back contacts of the proposed solar cells.

HTL Material	Metal Back Contacts PCE (%)				
	Al	Au	Cu	Mo	Se
**CFTS**	22.23	27.87	27.87	27.87	27.87
**CZTSe**	-	24.04	23.90	26.22	26.22
**CNTS**	-	24.64	24.37	20.64	24.81
**CZTSSe**	-	25.22	22.16	18.12	25.25

**Table 5:** Comparison of the theoretical and experimental data from different solar cell configurations.

Cell Configuration	Nature	V_oc_ (V)	J_sc_ (mA cm^−2^)	FF (%)	PCE (%)	Ref
**TFSA-GR/MoS_2_/P3HT:PCBM/Al**	Experimental	0.58	10.01	60.94	3.56	[97]
**FTO/ZnO/ZrS_2_/MoS_2_/CuO_2_/Au**	Simulation	0.84	36.02	68.54	20.64	[98]
**AZO/ZrS_2_/MoS_2_**	Simulation	0.57	34.02	71.35	14.13	[99]
**FTO/TiO_2_/MoS_2_/CFTS/Ag**	Simulation	1.93	34.52	38.79	25.86	This study
**FTO/TiO_2_/MoS_2_/CZTSe/Ag**	Simulation	0.80	29.90	85.69	20.56	This study
**FTO/TiO_2_/MoS_2_/CZNTS/Ag**	Simulation	0.70	31.67	59.84	13.29	This study
**FTO/TiO_2_/MoS_2_/CZTSSe/Ag**	Simulation	0.37	35.34	74.95	9.86	This study

## Data Availability

The original contributions presented in this study are included in the article. Further inquiries can be directed to the corresponding authors.

## Supplementary Materials

The following supporting information can be downloaded at: https://www.mdpi.com/article/10.3390/nano14242016/s1, Figure S1: Effect of thickness variation of HTL on PCE, FF, Jsc, and Voc parameters: (a) CFTS, (b) CZTSe, (c) CNTS, and (d) CZTSSe-based devices; Figure S2: Effect of thickness variation of ETL on PCE, FF, Jsc, and Voc parameters: (a) CFTS, (b) CZTSe, (c) CNTS, and (d) CZTSSe-based devices.
