# Supplementary material for: A Numerical Simulation Study of the Impact of Kesterites Hole Transport Materials in Quantum Dot-Sensitized Solar Cells Using SCAPS-1D
Source: Nanomaterials (Basel). 2024 Dec 15;14(24):2016. doi: 10.3390/nano14242016 (PMC11728719; doi:10.3390/nano14242016)
Supplement: Supplementary file 1 [file nanomaterials-14-02016-s001.zip › nanomaterials-3352389-supplementary.pdf]

A numerical simulation study of the impact of kesterites hole transport materials in quantum dot-sensitized solar cells using SCAPS-1D

Sindisiwe Jakalase[1, 2], Azile Nqombolo[2], Edson L. Meyer[1], Adedoyin M. Agoro[1,2] and Nicholas Rono*[1]

[1]Fort Hare Institute of Technology, University of Fort Hare, Private bag X1314, Alice 5700, South Africa,

[2]Department of Chemistry, University of Fort Hare, Private bag X1314, Alice 5700, South Africa.

*Corresponding author email: rononicholas21@gmail.com; Tel.: +27-738942558

Supplementary information

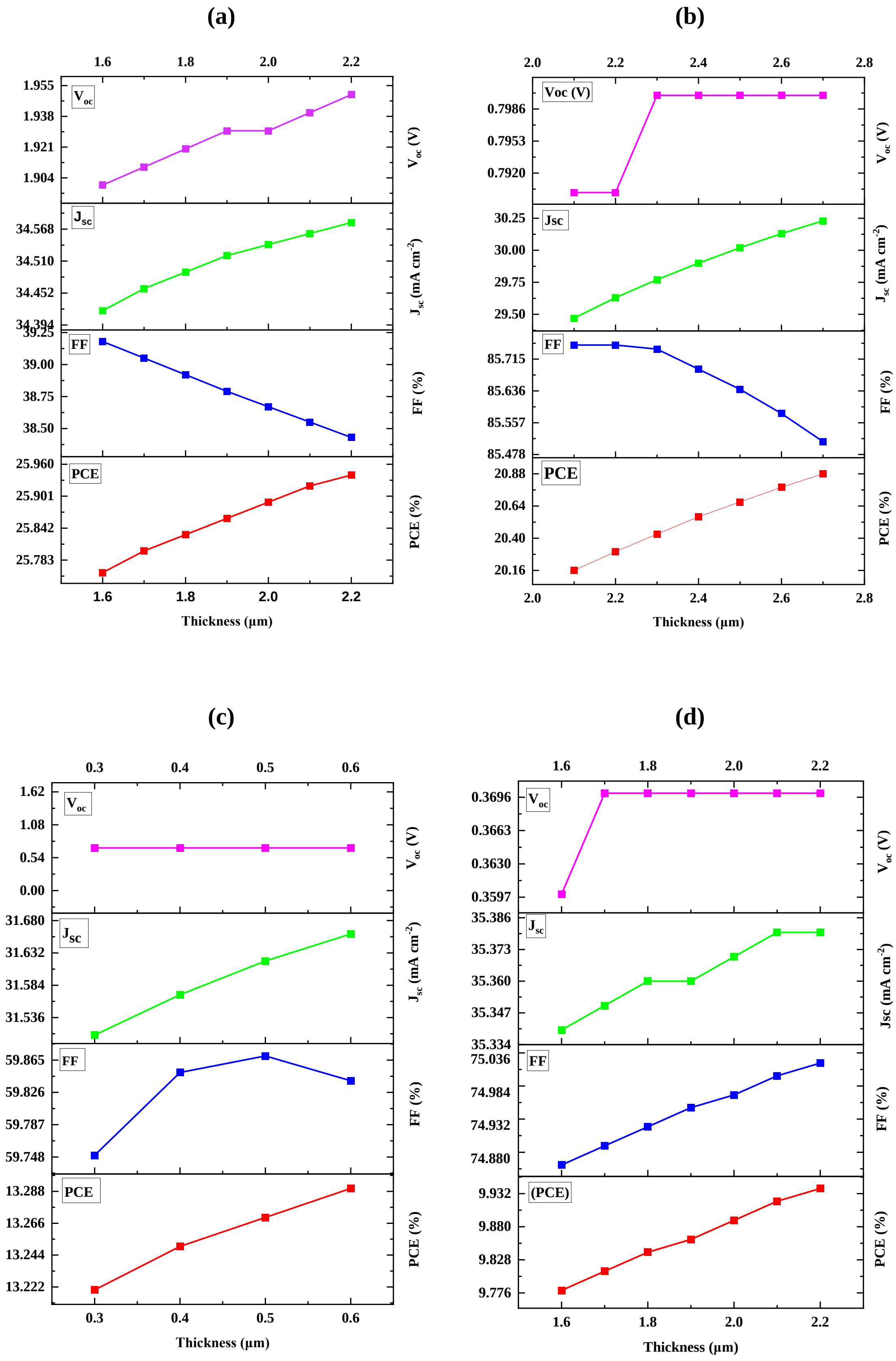

Figure S1: Effect of thickness variation of HTL on PCE, FF, J_{sc} and V_{oc} parameters **a**) CFTS, **b**) CZTSe, **c**) CNTS, and **d**) CZTSSe-based devices

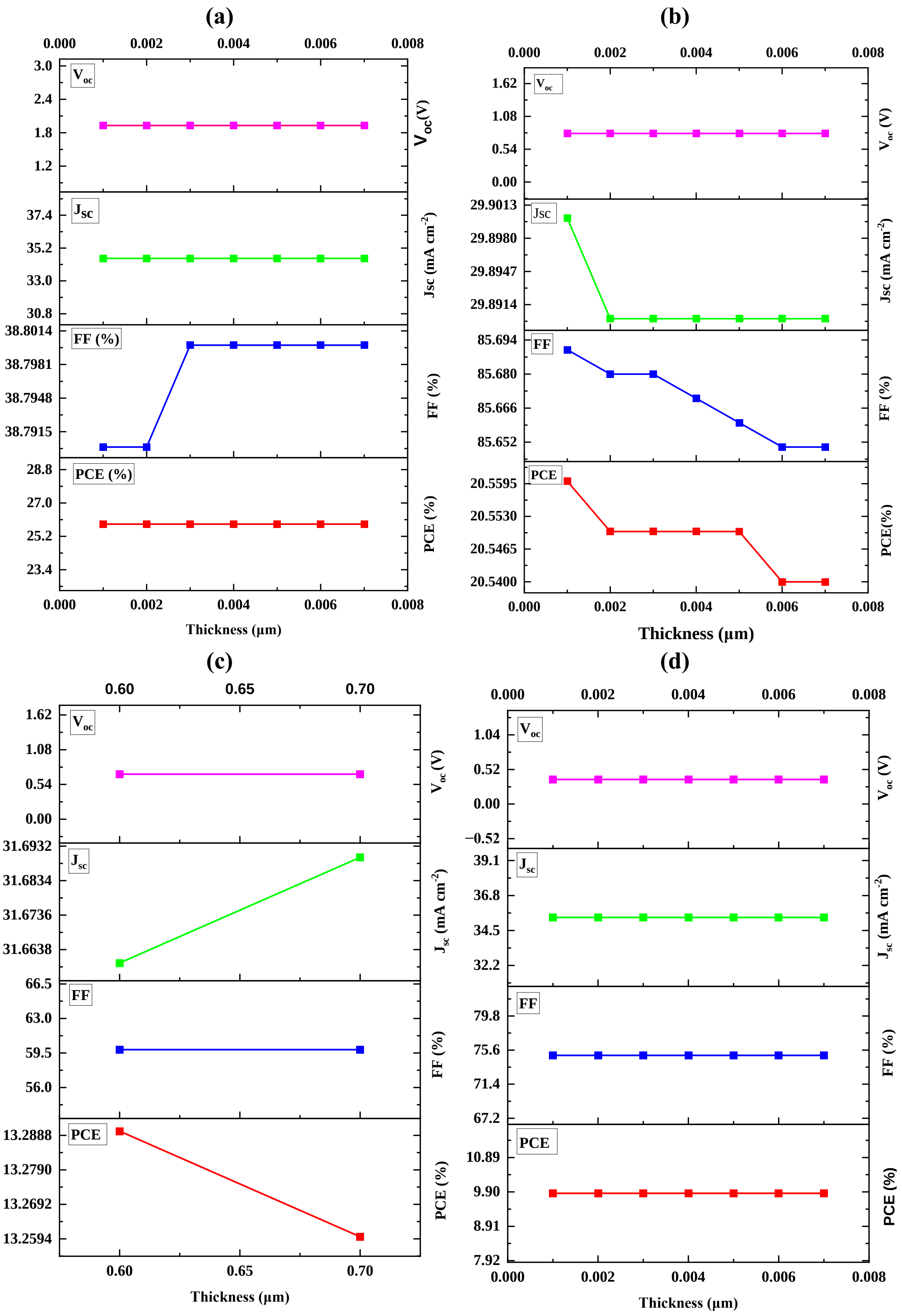

Figure S2: Effect of thickness variation of ETL on PCE, FF, J_{sc} and V_{oc} parameters **a**) CFTS, **b**) CZTSe, **c**) CNTS, and **d**) CZTSSe-based devices
